# Influence of the carbohydrate-binding module on the activity of a fungal AA9 lytic polysaccharide monooxygenase on cellulosic substrates

**DOI:** 10.1186/s13068-019-1548-y

**Published:** 2019-09-03

**Authors:** Amani Chalak, Ana Villares, Celine Moreau, Mireille Haon, Sacha Grisel, Angélina d’Orlando, Isabelle Herpoël-Gimbert, Aurore Labourel, Bernard Cathala, Jean-Guy Berrin

**Affiliations:** 1 Biopolymères Interactions Assemblages, INRA, Nantes, France; 2 Biodiversité et Biotechnologie Fongiques, UMR1163, INRA, Aix Marseille Université, Marseille, France

**Keywords:** Filamentous fungi, Cellulose, LPMO, CBH, CBM, Microscopy

## Abstract

**Background:**

Cellulose-active lytic polysaccharide monooxygenases (LPMOs) secreted by filamentous fungi play a key role in the degradation of recalcitrant lignocellulosic biomass. They can occur as multidomain proteins fused to a carbohydrate-binding module (CBM). From a biotech perspective, LPMOs are promising innovative tools for producing nanocelluloses and biofuels, but their direct action on cellulosic substrates is not fully understood.

**Results:**

In this study, we probed the role of the CBM from family 1 (CBM1) appended to the LPMO9H from *Podospora anserina* (*Pa*LPMO9H) using model cellulosic substrates. Deletion of the CBM1 weakened the binding to cellulose nanofibrils, amorphous and crystalline cellulose. Although the release of soluble sugars from cellulose was drastically reduced under standard conditions, the truncated LPMO retained some activity on soluble oligosaccharides. The cellulolytic action of the truncated LPMO was demonstrated using synergy experiments with a cellobiohydrolase (CBH). The truncated LPMO was still able to improve the efficiency of the CBH on cellulose nanofibrils in the same range as the full-length LPMO. Increasing the substrate concentration enhanced the performance of *Pa*LPMO9H without CBM in terms of product release. Interestingly, removing the CBM also altered the regioselectivity of *Pa*LPMO9H, significantly increasing cleavage at the C1 position. Analysis of the insoluble fraction of cellulosic substrates evaluated by optical and atomic force microscopy confirmed that the CBM1 module was not strictly required to promote disruption of the cellulose network.

**Conclusions:**

Absence of the CBM1 does not preclude the activity of the LPMO on cellulose but its presence has an important role in driving the enzyme to the substrate and releasing more soluble sugars (both oxidized and non-oxidized), thus facilitating the detection of LPMO activity at low substrate concentration. These results provide insights into the mechanism of action of fungal LPMOs on cellulose to produce nanocelluloses and biofuels.

## Background

Cellulose is the most abundant biopolymer on Earth and one of the main sources of renewable carbon [1]. Huge effort is being invested in the development of biofuels made from cellulosic biomass feedstocks, known as second-generation biofuels [2]. In parallel, nanomaterials such as nanofibers and nanocrystals are being isolated from wood and agricultural resources by mechanical and/or chemical treatments, offering unique properties with a wide range of applications (paper, pharmaceutical, cosmetics and food industries) [3–5]. The hierarchical complexity and recalcitrance of cellulose create a need to process it via innovative “green” pretreatments to address pressing global challenges and environmental concerns.

In nature, cellulose degradation is mainly achieved by filamentous fungi, which secrete complementary hydrolytic and oxidative activities. In contrast to known cellulases, which are hydrolytic enzymes, lytic polysaccharide monooxygenases (LPMOs) degrade cellulose via an oxidative mechanism [6–8] involving molecular oxygen or hydrogen peroxide and redox-active molecules acting as electron donors [9, 10]. LPMO-catalyzed cleavage leads to oxidation of one of the carbons in the scissile β-1,4-glycosidic bonds, i.e., oxidation of C1 and/or C4 of the glucose units, leading to carboxylic acid and/or keto functions at the cellulose surface [9, 11, 12]. LPMOs are widespread in the fungal kingdom, with five families of LPMOs (AA9, AA11, AA13, AA14, AA16) described in the CAZy database (www.cazy.org) [13, 14]. All characterized LPMOs that belong to the AA9 family are able to oxidatively cleave cellulose [15–18], and recent studies have focused on their use to defibrillate cellulose and facilitate the production of nanocelluloses [19–21].

The ascomycete *Podospora anserina* has been studied for its impressive array of CAZymes involved in both cellulose and hemicelluloses breakdown, making it a model of choice to better understand the enzymatic deconstruction of plant biomass [22, 23]. Its genome encodes 33 AA9 LPMOs (*Pa*LPMO9), eight of which contain a family 1 carbohydrate binding module (CBM1)-targeting cellulose. In the secretomes of *P. anserina* after growth on biomass, seven AA9 LPMOs were identified, five of which present a CBM1 [24]. Biochemical characterization of these enzymes showed various degrees of activity on cellulose, with higher total release of oxidized oligosaccharides from cellulose for *Pa*LPMO9A, *Pa*LPMO9E and *Pa*LPMO9H, all of which harbor a CBM1 module [17, 18]. *Pa*LPMO9H was then further investigated for its capacity to disrupt cellulose fibers [19] and was shown to cleave mixed-linkage glucans, xyloglucan and glucomannan [25], and oligosaccharides [18]. Mass spectrometry analysis of the released products revealed that *Pa*LPMO9H catalyzes C4 oxidative cleavage of mixed-linkage glucans and mixed C1/C4 oxidative cleavage of cellulose, glucomannan and xyloglucan [18, 25].

As stated earlier for *P. anserina*, the expansion in genes encoding AA9s has been observed in many fungal genomes. This gene multiplicity raises the question of the functional relevance at the organism level, i.e., functional redundancy or functional diversification and/or adaptations to substrate. Modular AA9 LPMOs bearing a CBM1 at their C-terminus are often predominantly secreted by filamentous fungi under lignocellulolytic conditions [26], but the role of these modules attached to LPMOs is not clearly established.

The roles of CBMs in glycoside hydrolase function have been widely explored (see [27] for review). Indeed, many glycoside hydrolases that attack the plant cell wall contain non-catalytic CBMs, which were first identified in cellulases [28]. CBMs are grouped into three types: type-A CBMs bind crystalline ligands while types B and C bind internal or terminal regions of polysaccharides, respectively. CBM1 is a type-A CBM, which binds crystalline substrates using a planar surface [29]. CBMs not only target the enzymes to their substrates to promote catalysis [30, 31], but sometimes they can also modulate enzyme specificity [32]. CBMs are devoid of catalytic activity, but some studies suggest they play a role in the amorphization of cellulose through non-hydrolytic disruption of the crystalline structure of cellulose [33, 34]. CBM1 appended to AA9 LPMOs may influence substrate binding, enzyme activity and/or regioselectivity, but the data are scarce and reported observations are contradictory. For instance, deletion of the CBM1 of NcLPMO9C had no effect on the degradation of PASC [35], whereas removal of the natural CBM from cellulose-active bacterial LPMOs abolished their activity [36].

Here, we investigate the role played by the CBM1 module to the cellulolytic activity of a fungal AA9 LPMO. *Pa*LPMO9H was chosen as our model enzyme. The CBM1 module was truncated, and enzymatic activity was investigated using complementary approaches to examine the release of soluble products and the cellulosic fibers themselves.

## Results

### Production of PaLPMO9H with and without CBM1

**Fig. 1:**
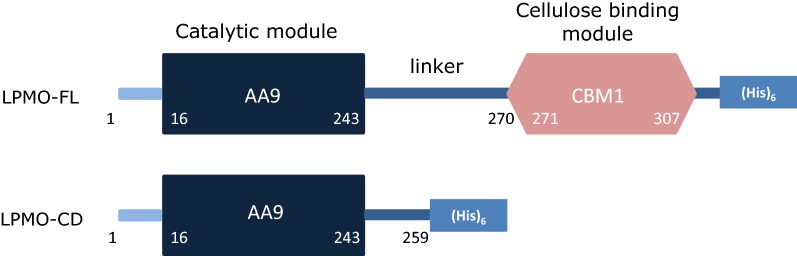
Schematic representation of the enzymes used in this study. LPMO-FL (full-length) and LPMO-CD (catalytic domain) with amino-acid numbering of the limits of each domain

To gain insight into the contribution of the CBM1 to the catalytic function of AA9 LPMOs, we selected *Pa*LPMO9H based on the previous biochemical analyses [18, 19, 25]. *Pa*LPMO9H is a modular enzyme with two domains containing an N-terminal catalytic AA9 domain (16–243) and a C-terminal CBM1 domain (271–307) (Fig. 1). These two domains are connected through a serine/threonine/asparagine-rich linker comprising 27 amino acid residues. When the *Pa*LPMO9H enzyme was truncated right after the catalytic module at position 244, we were unable to successfully produce the corresponding recombinant protein in *P. pastoris* (data not shown). Therefore, we decided to leave 16 amino acid residues of the linker to promote production of the recombinant enzyme. Using this strategy, we successfully produced the CBM1-free *Pa*LPMO9H enzyme truncated at position 259. In the rest of the study, the *Pa*LPMO9H with the CBM1 is named LPMO-FL (full-length), and the *Pa*LPMO9H without the CBM1 is named LPMO-CD (catalytic domain). As expected, deletion of the CBM1 decreased the enzyme’s molecular mass from ~ 38 kDa (LPMO-FL) to ~ 33 kDa (LPMO-CD). Apparent molecular mass was still slightly higher than theoretical molecular mass (25.7 kDa) due to predicted O- and N-glycosylations (Additional file 1: Figure S1). LPMOs are copper-dependent enzymes, which makes it crucial to check the correct copper protein loading. The amount of copper in each enzyme was quantified using inductively coupled plasma mass spectrometry (ICP-MS). Both enzymes were equally loaded with ~ 1 copper atom per molecule (i.e. 10.3 and 10.8 µM of Cu^2+^ for 10 µM of LPMO-FL and LPMO-CD, respectively).

### Absence of CBM1 alters LPMO cellulolytic activity at low substrate concentration

**Fig. 2:**
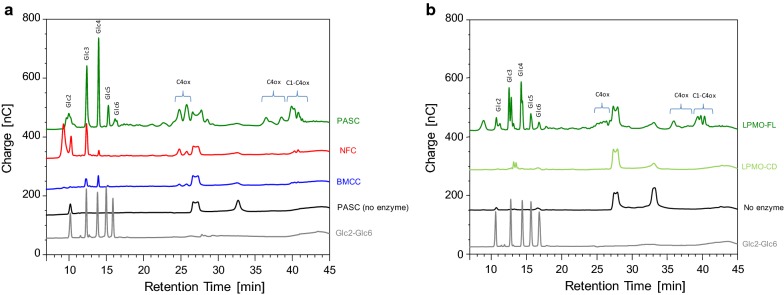
Analysis of soluble degradation products. **a** Products generated by LPMO-FL upon degradation of 0.1% PASC, NFC or BMCC with 4.4 µM of LPMO in the presence of 1 mM of l-cysteine, at 50 °C for 16 h. **b** Analysis of soluble degradation products generated by LPMO-FL and LPMO-CD upon degradation of 0.1% PASC with 4.4 µM of LPMO in the presence of 1 mM of l-cysteine, at 50 °C for 4 h

The action of LPMO-FL was first evaluated on three different types of cellulose, i.e., phosphoric acid-swollen cellulose (PASC), nanofibrillated cellulose (NFC), and bacterial microcrystalline cellulose (BMCC) (Fig. 2a). As previously shown, LPMO-FL released both C4-oxidized (C4ox) and non-oxidized oligosaccharides from PASC [18]. However, using NFC as a substrate led to less products released, and using a more recalcitrant crystalline substrate (BMCC) led to barely detectable products (Fig. 2a). We then compared the action of both LPMO-FL and LPMO-CD by measuring the release of sugars from PASC (Fig. 2b). LPMO-FL released higher amounts of soluble sugars (both oxidized and non-oxidized oligosaccharides) compared to LPMO-CD where soluble sugars were barely detectable (Fig. 2b).

Since LPMO-FL is active on soluble oligosaccharides [18], we investigated the activity of both LPMO-FL and LPMO-CD on cellohexaose as substrate (Additional file 1: Figure S2). A time-course analysis revealed that both enzymes were able to cleave cellohexaose, leading mainly to Glc3 and Glc4 non-oxidized products and C4-oxidized products. Although LPMO-FL showed slightly better activity than LPMO-CD over the 24-h time-course, the observed cleavage of cellohexaose by LPMO-CD confirms that the enzyme lacking the CBM1 module is still functional.

LPMO-FL and LPMO-CD binding to PASC, BMCC and NFC was assessed in the absence of reductant using pull-down assays to assess the impact of the CBM1 (Additional file 1: Figure S3). LPMO-FL was observed in the bound fraction of all three cellulosic substrates tested. However, in the absence of CBM1, there were no bands corresponding to LPMO-CD in the bound fraction. Therefore, the CBM1 promotes LPMO binding to the cellulosic substrates.

### Combined action of LPMO-FL and LPMO-CD with a cellobiohydrolase

**Fig. 3:**
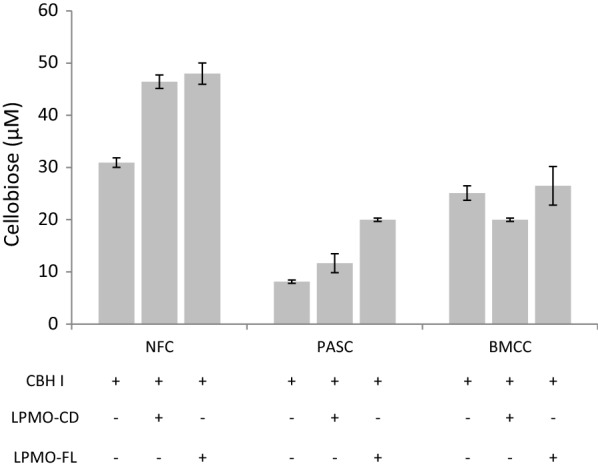
Combined action of LPMO-FL and LPMO-CD with a cellobiohydrolase (CBH). The cellobiose released (in µM) from the three cellulosic substrates NFC, PASC and BMCC was quantified using ion chromatography

To assess the impact of LPMO-CD on cellulosic substrates, we assayed LPMO-FL and LPMO-CD enzymes in combination with the reducing end-acting cellobiohydrolase (family GH7 CBH-I) from *T. reesei*. Cellulosic substrates were sequentially pretreated with either LPMO-FL or LPMO-CD before addition of the CBH-I enzyme. As both LPMOs and CBH-I act on soluble substrates, we implemented an LPMO post-treatment washing step to assess the impact of LPMO treatment only on the insoluble fibers. LPMO pretreatment was beneficial on PASC and NFC but had no visible effect on the crystalline substrate BMCC (Fig. 3). Pretreatment with either LPMO-CD or LPMO-FL increased cellobiose release from NFC substrate by approximately 30%. However, LPMO-FL pretreatment was more efficient on PASC substrate (60% increase in cellobiose production) compared to the LPMO-CD. Taken together, these results show that neither of the two LPMOs targets the crystalline fraction of cellulose. We believe that both LPMOs target amorphous regions thus facilitating CBH-I activity on crystalline cellulose. Moreover, under these experimental conditions the presence of the CBM1 module is not strictly required for LPMO action.

### Increasing substrate concentration reduces the need for the CBM1

**Fig. 4:**
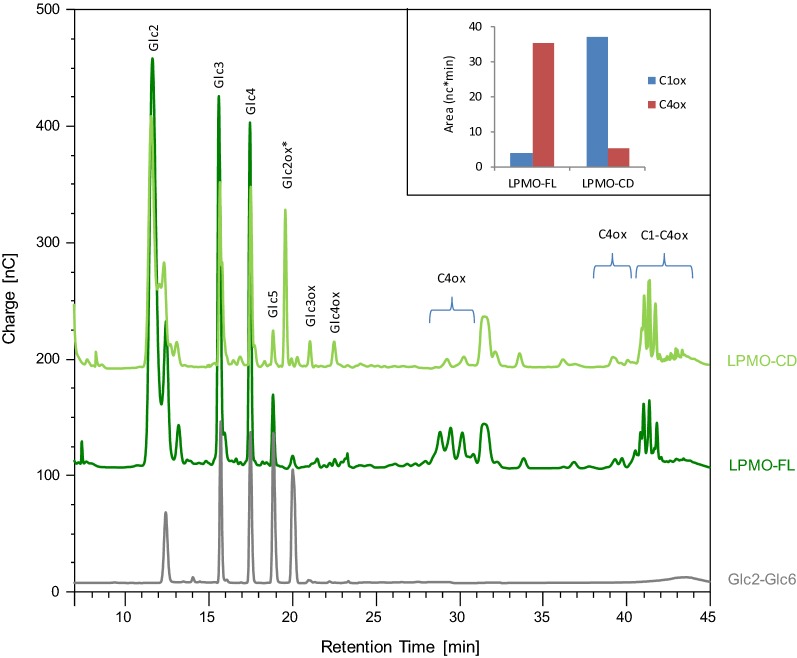
Analysis of degradation products generated by LPMO-FL and LPMO-CD. HPAEC chromatograms of the oligosaccharides released upon degradation of PASC [1% (w/v)] with 4.4 µM of LPMO in the presence of 1 mM of l-cysteine, at 50 °C for 16 h. The sum of C1-oxidized (C1ox) and C4-oxidized (C4ox) oligosaccharides is indicated in the inset. *Glc2ox co-eluted with Glc6

The next step was to assess whether substrate concentration has an influence on the activity of the enzymes. We increased the substrate concentration to 1% (w/v) to foster the probability of enzyme–substrate interactions in a CBM-free context. At high substrate concentration, soluble sugars released by LPMO-CD became detectable and were in the same range as soluble sugars released by LPMO-FL from PASC (Fig. 4). Interestingly, C1-oxidized (C1ox) products (Glc2ox-Glc4ox), which were barely detectable using LPMO-FL, were abundantly released by LPMO-CD (Fig. 4). The C4-oxidized products eluting at around 30 min were less abundant whereas the C1/C4-oxidized products eluting between 41 and 42 min were slightly increased. The absence of the CBM induced a modification of the regioselectivity pattern of the enzyme (Fig. 4).

### Impact of LPMO on the insoluble fraction

**Fig. 5:**
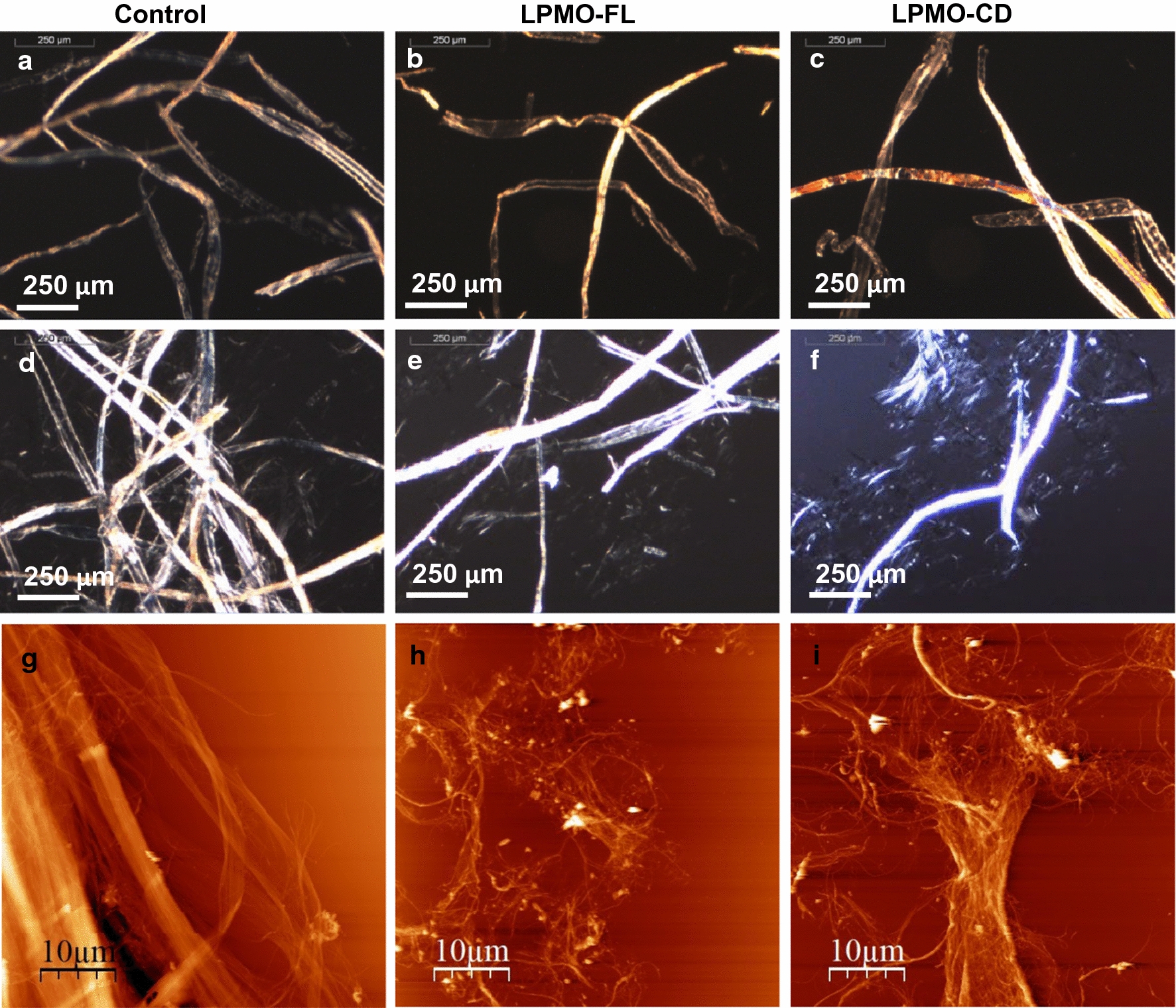
Morphology of LPMO-treated kraft fibers. Optical microscopy images of kraft fibers before (**a**–**c**) and after (**d**–**f**) mechanical dispersion for control samples (**a**, **d**), LPMO-FL-treated fibers (**b**, **e**) and LPMO-CD-treated fibers (**c**, **f**). AFM topographical images after LPMO treatment and dispersion for control kraft fibers (**g**), LPMO-FL-treated fibers (**h**) and LPMO-CD-treated fibers (**i**)

In an effort to gain more insight into the role of CBM on the action of LPMOs, we evaluated the changes in morphology of kraft fibers in response to incubation with LPMO. First, we investigated fiber structure using optical microscopy. Original kraft fibers are tens of micrometers in diameter and around 1 mm long (Fig. 5a). After LPMO treatment, there were no visible changes in physical appearance of the fibers, i.e., fibrous morphology or dimensions, in LPMO-FL (Fig. 5b) and LPMO-CD-treated samples (Fig. 5c). As previously described [19], the action of LPMOs alone does not produce a noticeable disintegration of kraft fibers, similarly to the action of cellulases [37–39]. Therefore, after LPMO treatment, fibers were mechanically dispersed then subjected to a short ultrasound treatment. Dispersion revealed the effect of LPMO on kraft fibers. Control samples showed some slight defibrillation whereas both LPMO-treated samples showed clear cell wall delamination (Fig. 5d–f). Both LPMO-FL and LPMO-CD seemed to create weak points within the fiber that facilitated the mechanical disintegration. To get a better picture of the effect of the LPMOs, we used atomic force microscopy (AFM) to analyze samples (Fig. 5g–i). Topography images showed the presence of large fibers in control samples and a clear dissociation in LPMO-treated samples. LPMO-FL produced fibrillation of kraft fibers, forming an entangled network of ~ 5 nm-diameter nanofibrils. LPMO-CD also produced a network of disintegrated fibers, but with thicker fiber bundles. Comparing the appearance of the fibers treated with LPMO-FL or LPMO-CD against controls, it is evident that both enzymes influence the cohesion and architecture of the fibers, making them more prone to the mechanical forces caused by the dispersion. Based on AFM images, both LPMOs reduced fiber cohesion, but the presence of CBM appeared to enable LPMO-FL to defibrillate cellulose.

## Discussion

The functional relevance of CBMs and their contribution to the activity of the LPMO enzymes have already been investigated [36, 40], but in several cases, analysis surprisingly found modest and contradictory effects on enzyme activity. The role of CBMs appended to glycoside hydrolases has been explored in depth [27], and it is generally acknowledged that the presence of CBMs increases the concentration of proteins at the surface of the substrate, thus increasing the activity of the enzyme [41]. Removal of the CBM attached to cellulases dramatically decreases the activity on insoluble substrates but not on soluble substrates [37, 42, 43]. A similar pattern was observed here with *Pa*LPMO9H, as loss of the CBM dramatically affected the release of soluble sugars from cellulose whereas activity was retained on soluble cellooligosaccharides. However, when the substrate concentration of cellulose was increased, the lack of CBM did not seem to impede the action of *Pa*LPMO9H (LPMO-CD), and soluble products were detected in the same range as the full-length enzyme. A similar pattern of action was observed with cellulases where a reduced amount of water counterbalanced the need for CBMs [44]. Our results are in line with the hypotheses drawn by Courtade et al. [45] on a cellulose-active AA10 LPMO. Indeed, multiple cleavages are needed at the cellulose surface to release enough soluble products that can then be detected by ion chromatography. Here, we observed that the CBM1 appended to an AA9 LPMO promotes binding to cellulose and anchors the enzyme to the substrate, facilitating multiple localized cleavages. Conversely, AA9 LPMOs lacking CBM1 only weakly bind to cellulose and may thus have a more random action on cellulose, thus limiting the number of localized cleavages and therefore the release of soluble cellooligosaccharides (<Glc6). This hypothesis is further supported by the synergistic effect with a CBH observed for both enzymes (with and without CBMs) on cellulose fibers and their capacity to defibrillate cellulose. Note, however, that although LPMO-FL was able to bind crystalline cellulose, it showed no detectable activity, meaning that *Pa*LPMO9H may target less-organized regions of cellulose, as already hypothesized in [19].

Surprisingly, CBM deletion was found to modify the regioselectivity pattern of the enzyme. The regioselectivity pattern was also modified when aromatic residues at the substrate-binding interface of *Hj*LPMO9A were mutated [40], but removal of the *Hj*LPMO9A CBM did not alter the regioselectivity of the enzyme even though the effect of mutations was increased in a CBM-free context [40]. It seems that altering the mode of LPMO-to-substrate binding may slightly modify the position of the enzyme at the cellulose surface, thus generating a mixture of C1 and C4 cleavages. The fact that presence of the CBM may influence the regioselectivity of cellulose cleavage in LPMOs challenges the established pseudo-classification [46] that contains many exceptions, and raises questions as to the functional relevance of the C1 and/or C4 cleavage in LPMOs.

## Conclusions

Assays of LPMO activity based on detecting soluble products warrant careful assessment taking into account the nature and concentration of the substrate. More generally, from a microbial degradation perspective, the fact that filamentous fungi secreted a wide range of AA9 LPMOs with and without CBMs may be exploitable to promote degradation depending on the substrate consistency. From a biotech perspective, the presence of a CBM appended to LPMOs could be mobilized to select targets for cellulose degradation purposes. However, regarding cellulose defibrillation for nanocellulose production, more work is needed to pinpoint the influence of the CBM on the efficiency of LPMOs used in the process.

## Materials and methods

### Substrates

This study used several cellulosic substrates, representing either the crystalline, amorphous, or alternating crystalline and amorphous regions, or quasi-natural fibers like the kraft fibers. Phosphoric acid-swollen cellulose (PASC) was prepared as described previously [18]. Nanofibrillated cellulose (NFC) obtained via endoglucanase pretreatment followed by microfluidization was kindly provided by the Centre Technique du Papier (CTP, Grenoble, France). Bacterial microcrystalline cellulose (BMCC) was obtained from nata de coco cubes that were subjected to hydrochloric acid (2.5 N) hydrolysis at a temperature of 72 °C in three consecutive steps over a total time of 2 h, then separated by filtration and three centrifugation cycles at 10,000*g* for 10 min in which the acid supernatant was repeatedly replaced by water. Then, dialysis was done against distilled water. Bleached softwood kraft pulp was used as a substrate. Cellulose fibers were dispersed in 50 mM of sodium acetate buffer (pH 5.2) and stirred for 48 h prior to enzymatic assays [19].

### Recombinant production of LPMO enzymes

*Pa*LPMO9H (protein ID CAP 61476) was produced in *Pichia pastoris* as described in [18]. To produce *Pa*LPMO9H without CBM, the gene region coding for its amino acid sequence 1–259 (see Fig. 1) was amplified and inserted into the pPICZalphaA vector (Invitrogen, Cergy-Pontoise, France) using BstBI and XbaI restriction sites in-frame with the (His)_6_ tag. *P. pastoris* strain X33 and the pPICZalphaA vector are components of the *P. pastoris* Easy select expression system (Invitrogen). All media and protocols are described in the *Pichia* expression manual (Invitrogen). Recombinant expression plasmids were sequenced to check the integrity of the corresponding sequences.

Transformation of competent *P pastoris* X33 was performed by electroporation with *Pme*I-linearized pPICZalphaA recombinant plasmid as described in [30]. Zeocin-resistant *P. pastoris* transformants were then screened for protein production. The best-producing transformant was grown in 1 L of BMGY containing 1 mL L^−1^ of *Pichia* trace minerals 4 (PTM4) salts (2 g L^−1^ CuSO_4_·5H_2_O, 3 g L^−1^ MnSO_4_·H_2_O, 0.2 g L^−1^ Na_2_MoO_4_·2H_2_O, 0.02 g L^−1^ H_3_BO_3_, 0.5 g L^−1^ CaSO_4_·2H_2_O, 0.5 g L^−1^ CaCl_2_, 12.5 g L^−1^ ZnSO_4_·7H_2_O, 22 g L^−1^ FeSO_4_·7H_2_O, biotin 0.2 g L^−1^, H_2_SO_4_ 1 mL L^−1^) in flasks shaken at 30 °C in an orbital shaker (200 rpm) for 16 h to reach an OD_600_ of 2–6. Expression was induced by transferring cells into 200 mL of BMMY containing 1 mL L^−1^ of PTM4 salts at 20 °C in an orbital shaker (200 rpm) for another 3 days. Each day, the medium was supplemented with 3% (v/v) methanol.

### Enzyme purification

After harvesting cells by centrifugation (2700*g* for 5 min, 4 °C), the supernatant was adjusted to pH 7.8 just before purification, filtered on 0.22-µm filters (Millipore, Molsheim, France), and loaded onto a 5-mL HiTrap HP column (GE Healthcare, Buc, France) equilibrated with buffer A (Tris–HCl 50 mM pH 7.8, NaCl 150 mM, imidazole 10 mM) that was connected to an Äkta purifier 100 system (GE Healthcare). Each (His)_6_-tagged recombinant enzyme was eluted with buffer B (Tris–HCl 50 mM pH 7.8, NaCl 150 mM, imidazole 500 mM). Fractions containing recombinant enzymes were pooled and concentrated with a 10-kDa vivaspin ultrafiltration unit (Sartorius, Palaiseau, France) and filter dialyzed against sodium acetate buffer 50 mM, pH 5.2. The concentrated proteins were incubated overnight with an equimolar equivalent of CuSO_4_ in a cold room and buffer exchanged in 50 mM sodium acetate buffer pH 5.2 using extensive washing in a 10-kDa ultrafiltration unit to remove traces of CuSO_4_.

### Protein analysis

Proteins were loaded onto 10% Tris–glycine precast SDS-PAGE gels (BioRad, Marnes-la Coquette, France) and stained with Coomassie Blue. The molecular mass under denaturing conditions was determined with PageRuler Prestained Protein Ladder (Thermo Fisher Scientific, IL). The protein concentrations were determined by adsorption at 280 nm using a Nanodrop ND-2000 spectrophotometer (Thermo Fisher Scientific) with theoretical molecular masses and molar extinction coefficient derived from the sequences (49,640 and 39,545 M^−1^ cm^−1^ for LPMO-FL and LPMO-CD, respectively, measured at 280 nm in water).

### ICP-MS analysis

The ICP-MS analysis was performed as described in [47]. The samples were mineralized, then diluted in ultrapure water, and analyzed on an ICAP Q apparatus (Thermo Electron, Les Ulis, France). Copper concentration was determined using Plasmalab (Thermo Electron) software, at *m*/*z* = 63.

### Qualitative cellulose-binding assays

The reaction mixtures were carried out at 0.3% (w/v) insoluble substrate loading (BMCC; NFC; PASC) and 30 µg of proteins were added. The reactions were done in 50 mM sodium acetate buffer pH 5.2 in a final volume of 200 µL without any l-cysteine addition. The tubes were incubated on ice for 1 h with gentle mixing every 10 min. After centrifugation at 14,000*g* for 10 min, the supernatant (containing the unbound proteins) was carefully removed, then the polysaccharide pellets were washed twice (wash 1 and wash 2) by resuspending in buffer and centrifuged at 14,000*g* for 10 min. This step was repeated twice. The remaining pellet was finally resuspended in SDS-loading buffer without dye (with a volume equivalent to the unbound fraction removed) and boiled for 10 min to dissociate any bound protein. Unbound, wash 2 and bound fractions (45 µL supplemented with 5 µL of β-mercaptoethanol) were analyzed by SDS-PAGE to detect the presence or absence of the protein. The supernatant was recovered (supernatant 2: bound fraction), and 45 µL of supernatant 1 (unbound fraction), wash 2 and supernatant 2 (bound fraction) were analyzed by SDS-PAGE to detect the presence or absence of the protein. We ran a control sample without any substrate to compare the results.

### Enzymatic treatment of the substrates for the analysis of soluble sugars

All the cleavage assays (on a final volume of 300 μL) contained 0.1% (w/v) of substrate (PASC, BMCC, NFC), 4.4 µM of *Pa*LPMO9s, and 1 mM of l-cysteine, in 50 mM sodium acetate buffer pH 5.2. The enzymatic reactions were incubated in a thermomixer (Eppendorf, Montesson, France) at 50 °C and 850 rpm for 16 h. At the end of the reaction, the samples were boiled at 100 °C for 15 min and then centrifuged at 15,000*g* for 10 min to separate the soluble and insoluble fractions. Assays at 1% (w/v) PASC concentration were also done in the conditions mentioned earlier.

### Combined assays

The LPMO enzymatic assays were carried out sequentially with a cellobiohydrolase from *T. reesei* (CBH-I) as described in [48]. Assays were performed in a total volume of 800 µL containing 0.1% (v/w) cellulose in 50 mM pH 5.2 acetate buffer with 8 µg of LPMO enzyme and 1 mM l-cysteine. The samples were incubated in triplicate in a thermomixer (Eppendorf) at 45 °C and 850 rpm, for 24 h. The samples were then boiled for at least 10 min and centrifuged at 15,000*g* for 10 min. The supernatant was removed, and the remaining insoluble fraction of the substrate was washed twice in buffer. Hydrolysis by CBH-I (0.8 µg) was performed in 800 µL of 50 mM pH 5.2 acetate buffer for 2 h at 45 °C and 850 rpm. The soluble fraction was analyzed as described below.

### Analysis of oligosaccharides

Oxidized and non-oxidized cellooligosaccharides generated after LPMO action were analyzed by high-performance anion-exchange chromatography coupled with pulsed amperometric detection (HPAEC-PAD) (ThermoFischer Scientific, IL) using a CarboPac™ PA1 column (2 × 250 mm) and CarboPac™ PA1 guard column (2 × 50 mm) at a 0.25 mL min^−1^ flow rate as in [49]. Non-oxidized oligosaccharides were used as standards (Megazyme, Wicklow, Ireland).

### Enzymatic treatment of the softwood pulp for the analysis of the insoluble fibers

Kraft fibers (100 mg) were adjusted to pH 5.2 with sodium acetate buffer (50 mM) in a final reaction volume of 20 mL with 1 mM l-cysteine. Purified LPMO enzyme was added to the substrate at a final concentration of 1.6 µM. Enzymatic incubation was performed at 50 °C under mild agitation for 16 h. Samples were then dispersed with a Polytron PT 2100 homogenizer (Kinematica AG, Germany) for 3 min then ultrasonicated with a QSonica Q700 sonicator (20 kHz, QSonica LLC, Newtown, CT) at 350 W ultrasound power for 3 min. The reference sample was submitted to the same treatment but did not contain the LPMO enzyme.

### Optical microscopy

Kraft fibers (reference and LPMO-treated) were deposited onto a glass slide and observed under a BX51 polarizing microscope (Olympus France S.A.S.) with a 4× objective. Images (*N* ≥ 20) were captured by a U-CMAD3 camera (Olympus, Japan). The concentration of the fibers used was 2.5 g L^−1^ to visualize individual and separated fibers.

### Atomic force microscopy (AFM)

Fiber dispersions were diluted at 0.1 g L^−1^. Samples were dialyzed against ultrapure water (spectral por-; molecular porous membrane tubing 12–14 kDa) for 3 days to remove buffer, cysteine and released soluble sugars. They were later deposited onto mica substrates, allowed to settle for 45 min, and dried with Whatman filter paper. The final drying step was done in an incubator at 40 °C for 10 min before transfer to the AFM system. Topographical images on mica were registered by an Innova AFM system (Bruker). The images were collected in tapping mode under ambient air conditions (temperature and relative humidity) using a monolithic silicon tip (FESPA-V2) with a spring constant of 2.8 N m^−1^ and a nominal frequency of 75 kHz. Image processing was performed using WSxM 4.0 software. A series of reference images (between 3 and 11) were recorded to ensure the homogeneity of the sample.

## Supplementary information

**Additional file 1: Figure S1.** SDS-PAGE analysis of LPMO-FL and LPMO-CD purified enzymes. Ladder size is indicated in kDa. **Figure S2.** Time-course analysis and quantification of the Glc4 (plain lines) and Glc3 (dotted lines) released by LPMO-FL (triangles) and LPMO-CD (circles) acting on cellohexaose over a total period of 24 h. **Figure S3.** Qualitative cellulose binding assays. Binding of LPMO-FL and LPMO-CD to (a) PASC (0.3% (w/v)), (b) NFC (0.3% (w/v)) and (c) BMCC (0.3% (w/v)). Lane 1, control (no substrate); lane 2, unbound material; lane 3, wash 1; lane 4, bound fraction. Experiments were carried out on ice using 30 µg of proteins and 50 mM sodium acetate buffer pH 5.2 in a final volume 200 µL without added l-cysteine. Ladder size is given in kDa.

## Data Availability

The datasets used and/or analyzed during the current study are available from the corresponding author on reasonable request.

## Abbreviations

AA: auxiliary activity enzyme
BMCC: bacterial microcrystalline cellulose
CAZyme: carbohydrate-active enzyme
CBH: cellobiohydrolase
CBM: carbohydrate-binding module
C1ox: C1-oxidized oligos
C4ox: C4-oxidized oligos
Glc2: cellobiose
Glc3: cellotriose
Glc4: cellotetraose
Glc5: cellopentaose
Glc6: cellohexaose
HPAEC-PAD: high-performance anion-exchange chromatography coupled with amperometric detection
ICP-MS: inductively coupled plasma mass spectrometry
LPMO: lytic polysaccharide monooxygenase
LPMO-FL: LPMO full-length
LPMO-CD: LPMO catalytic domain
NFC: nanofibrillated cellulose
PASC: phosphoric acid-swollen cellulose
SDS-PAGE: sodium dodecyl sulfate-polyacrylamide gel electrophoresis
