# Supplementary material for: Influence of the carbohydrate-binding module on the activity of a fungal AA9 lytic polysaccharide monooxygenase on cellulosic substrates
Source: Biotechnol Biofuels. 2019 Sep 3;12:206. doi: 10.1186/s13068-019-1548-y (PMC6721207; doi:10.1186/s13068-019-1548-y)
Supplement: Supplementary file 1 — Additional file 1: Figure S1. SDS-PAGE analysis of LPMO-FL and LPMO-CD purified enzymes. Ladder size is indicated in kDa. Figure S2. Time-course analysis and quantification of the Glc4 (plain lines) and Glc3 (dotted lines) released by LPMO-FL (triangles) and LPMO-CD (circles) acting on cellohexaose over a total period of 24 h. Figure S3. Qualitative cellulose binding assays. Binding of LPMO-FL and LPMO-CD to (a) PASC (0.3% (w/v)), (b) NFC (0.3% (w/v)) and (c) BMCC (0.3% (w/v)). Lane 1, control (no substrate); lane 2, unbound material; lane 3, wash 1; lane 4, bound fraction. Experiments were carried out on ice using 30 µg of proteins and 50 mM sodium acetate buffer pH 5.2 in a final volume 200 µL without added l-cysteine. Ladder size is given in kDa. [file 13068_2019_1548_MOESM1_ESM.docx]

**Influence of the carbohydrate binding module on the activity of a fungal AA9 lytic polysaccharide monooxygenase on cellulosic substrates**

Amani Chalak, Ana Villares, Celine Moreau, Mireille Haon, Sacha Grisel, Angélina d’Orlando, Isabelle Herpoël-Gimbert, Aurore Labourel, Bernard Cathala, Jean-Guy Berrin

**Additional file**

**Figure S1:** SDS-PAGE analysis of LPMO-FL and LPMO-CD purified enzymes. The ladder size is indicated in kDa.

**Figure S2:** Time-course analysis and quantification of the Glc4 (plain lines) and Glc3 (dotted lines) released by LPMO-FL (triangles) and LPMO-CD (circles) acting on Glc6 (1mM) over a total period of 24 hours. Enzyme concentration was 1 µM.

**Figure S3:** Qualitative cellulose binding assays. Binding of LPMO-FL and LPMO-CD to (a) PASC (0.3% (w/v)), (b) NFC (0.3% (w/v)) and (c) BMCC (0.3% (w/v)). Lane 1, control (no substrate); lane 2, unbound material; lane 3, wash and lane 4, bound fraction. Experiments were carried out on ice using 30 µg of proteins and 50 mM sodium acetate buffer pH 5.2 in 200 µl final volume without addition of L-cysteine. The ladder size is indicated in kDa.
